# Titanium-based MAX-phase with sonocatalytic activity for degradation of oxytetracycline antibiotic

**DOI:** 10.1016/j.ultsonch.2022.106255

**Published:** 2022-12-05

**Authors:** Samira Haddadi, Alireza Khataee, Samira Arefi-Oskoui, Behrouz Vahid, Yasin Orooji, Yeojoon Yoon

**Affiliations:** a Research Laboratory of Advanced Water and Wastewater Treatment Processes, Department of Applied Chemistry, Faculty of Chemistry, University of Tabriz, Tabriz 51666-16471, Iran; b Рeoples’ Friendship University of Russia (RUDN University), 6 Miklukho-Maklaya Street, Moscow, 117198, Russian Federation; c Department of Chemical Industry, Technical and Vocational University (TVU), Tehran, Iran; d Department of Chemical Engineering, Tabriz Branch, Islamic Azad University, Tabriz, Iran; f College of Geography and Environmental Sciences, Zhejiang Normal University, Jinhua 321004, China; g Department of Environmental and Energy Engineering, Yonsei University, Wonju, Republic of Korea

**Keywords:** Ti_2_SnC MAX phase, Oxytetracycline, Sonocatalytic degradation, Emerging contaminants

## Abstract

In light of growing environmental concerns over emerging contaminants in aquatic environments, antibiotics in particular, have prompted the development of a new generation of effective sonocatalytic systems. In this study, a new type of nano-laminated material, Ti_2_SnC MAX phase, is prepared, characterized, and evaluated for the sonocatalytic degradation of oxytetracycline (OTC) antibiotic. A variety of identification analyses, including X-ray diffraction, scanning electron microscopy, energy-dispersive X-ray spectrometry, Brunauer-Emmett-Teller, and diffuse reflectance spectroscopy, were conducted to determine the physicochemical properties of the synthesized catalyst. By optimizing the operating factors, total degradation of OTC occurs within 120 min with 1 g L^-1^ catalyst, 10 mg L^-1^ OTC, at natural pH of 7.1 and 150 W ultrasonic power. The scavenger studies conclude that the singlet oxygen and superoxide ions are the most active species during the sonocatalytic reaction. Based on the obtained data and GC–MS analysis, a possible sonocatalytic mechanism for the OTC degradation in the presence of Ti_2_SnC is proposed. The catalyst reusability within eight consecutive runs reveals the proper stability of Ti_2_SnC MAX phase. The results indicate the prospect for MAX phase-based materials to be developed as efficient sonocatalysts in the treatment of antibiotics, suggesting a bright future for the field.

## Graphical abstract

**Figure:**
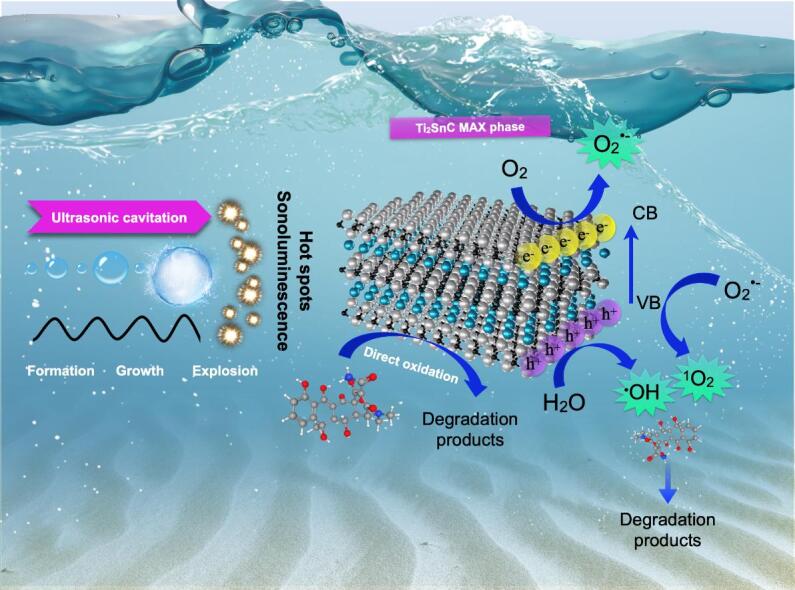

## 1 Introduction

A class of nanolaminate materials known as MAX phases has fueled the attention of researchers worldwide in recent years thanks to their unique features, which arise from their nanolaminated crystal structure [1], [2]. With hexagonal crystal symmetry, ternary M_n+1_AX_n_ phases are classified by their unique structural combination of metal-bonded A-layers (A-group elements) and XM_6_-octahedra layers (M is a transition metal, X is C and/or N). Each MAX phase features a different stacking form, with n being either 1 (211-type), 2 (312-type), or 3 (413-type) [3]. Elemental change to M, A, and/or X results in the formation of new MAX phases with better oxidation, strength and self-healing capabilities, and more importantly, the formation of new ordered structures [4]. The MAX phases are distinguished by the weaving properties of two completely different classes of materials: ceramics and metals [5]. These properties include high electrical and thermal conductivity, strength, and low density, which lend themselves to both classes [6], [7]. The MAX phases have scarcely been examined as catalysts, particularly in regard to sono/photocatalysis [8]. Nevertheless, their catalytic performance appraisal is increasing. As for water treatment applications, the MAX phases and their composites have so far been used in membranes [9], photocatalysis [10], and sonoctalysis [11].

Emerging contaminants have become a topic of growing interest in wastewater treatment. Antibiotics are just one of the key emerging contaminants that fall under the heading of pharmaceuticals [12]. The oxytetracycline (OTC), an antibiotic belonging to the tetracycline family, is among the most commonly prescribed and toxic antibiotics [13], [14]. Thus, human excrement, pharmaceutical effluents, as well as other human activities regularly contaminate the aquatic environment with OTC [15]. As a hydrophilic chemical that contains durable naphthalene rings, OTC is difficult to remove from wastewater using traditional and biological water treatment procedures [16]. Considering the extensive use, chemical stability, and high concentration of OTC in water bodies, it represents a danger to ecosystem health as well as the potential to develop antibiotic-resistant bacteria and diseases [17], [18], [19], [20]. Therefore, its water remediation is essential for the health of humans, plants, and terrestrial and marine animals.

Through advanced oxidation processes (AOPs), persistent water pollutants have been successfully removed [21]. Among various AOPs, sonocatalysis is increasingly being employed as a viable approach for degrading organic contaminants without generating secondary waste. Hence, it offers several advantages, including operational convenience, safety, eco-friendliness, and high decomposition efficiency [22]. By applying ultrasound (US) waves to a semi-conducting catalyst, reactive species are generated by acoustic cavitation and sonoluminescence, which accelerates catalysis. Through the addition of semiconducting materials as sonocatalysts, more nucleation sites for cavitation are provided for ultrasonic degradation of pollutants [23]. Eventually, the cavitation bubbles collapse and form electron–hole pairs on the semiconductor surfaces by the released energy or sonoluminescence emission, which results in the generation of reactive oxygen species (ROS) [24].

In this research, we outline the reactive sintering-based synthesis of the Ti_2_SnC MAX phase and its subsequent involvement in the sonocatalytic degradation of OTC. Analyzing the as-prepared catalyst’s characteristics was carried out in accordance with field-emission scanning electron microscope (FESEM), high-resolution transmission electron microscopy (HRTEM), X-ray diffraction (XRD), X-ray photoelectron spectroscopy (XPS), Brunauer–Emmett–Teller (BET), ultraviolet–visible diffuse reflectance spectroscopy (UV-DRS), energy-dispersive X-ray spectroscopy (EDX), and dot mapping technique analyses. In an effort to optimize the system, a variety of parameters were also investigated in relation to the pollutants degradation rate. The factors include the sonocatalyst dosage, the initial solution pH, the initial contaminant concentration, and the suppressive role of a variety of radical and nonradical scavengers. Moreover, the applicability of the optimal Ti_2_SnC sonocatalytic system for the elimination of multiple organic contaminants, such as Rifampin (RIF), Levofloxacin (LEV), and Acid blue 7 (AB7), was assessed. Data from a gas chromatograph mass spectrometer (GC–MS) provided to infer an ostensible mechanism for the OTC decomposition. Also, the reusability of the catalyst and its performance in real water samples were evaluated. To the extent of our knowledge, this research is the first to evaluate the Ti_2_SnC MAX phase's capability as a potent sonocatalyst for the decontamination of pharmaceutically contaminated water.

## 2 Materials and methods

### 2.1 Materials

Titanium (Ti) and tin (Sn) powders with a purity of 99.99 % and a mesh size of 1000 were acquired from STNMT Co., Ltd. Also, supplier of the graphite powder (C), which had a purity of 99.99 %, was Aladdin Reagent Co., Ltd. The following chemicals were purchased from the Merck Company (Germany)

hydrochloric acid (HCl), sodium hydroxide (NaOH), ethanol (C_2_H_5_OH), ethylenediaminetetraacetic acid (C_10_H_16_N_2_O_8_), l-ascorbic acid (C_6_H_8_O_6_), l-histidine (C_8_H_13_N_3_O_4_), furfuryl alcohol (C_5_H_6_O_2_), O-phenylenediamine (C_6_H_8_N_2_), dichloromethane (CH_2_Cl_2_), *N*-Methyl-2-pyrrolidone (NMP), and sodium sulfate (Na_2_SO_4_). Analytical grade materials were employed in this work without additional purification; OTC, RIF, LEV, and AB7 were obtained from RAZAK Pharmaceutical Co., Iran, Hakim Pharmaceutical Co., Iran, Abidi Pharmaceuticals Co., Iran, and Shimi Boyakhsaz Co., Iran, respectively.

### 2.2 Ti_2_SnC MAX phase synthesis

Approach to synthesize the Ti_2_SnC structure using the customized a reactive sintering [25], [26] method involves the following steps: planetary ball mill apparatus (ball: material = 10:1) was applied to mix titanium, graphite and tin powders with the molar ratio of Ti: Sn: C = 2:1.2:1 for 12 h at the revolution speed of 350 rpm. Under pressure of 250 MPa, the mixed powder was compressed into a disc. The as-prepared cake was introduced to an argon-gas-powered tube furnace with 100 SCCM air flow. The sample was subjected to a nonlinear stepped sintering with alteration of heating rates 1–10 °C min^-1^ up to 1200 °C. Once the material was cooled, it was grinned and sieved to yield the Ti_2_SnC powder. The purity of so-synthesized composite was increased by acid wash [27].

### 2.3 Analytical techniques

For morphological and elemental composition examinations, the FE-SEM images and the EDX spectra were collected using a Tescan Mira3 microscope (Czech Republic) at an acceleration voltage of 15 kV. By exposing the sample to Cu K radiation at 40 kV and 100 mA, the XRD patterns were obtained on a powder X-ray diffractometer (Siemens, Germany). To record the HRTEM pictures, a JEM-2100 Plus electron microscope was used (JEOL, Japan). The Thermo Scientific Escalab 250 Xi Plus XPS spectrometer (UK) was used in order to carry out the XPS measurements. By analyzing the N_2_ adsorption and desorption isotherms at 77 Kelvin using the Belsorp Mini II (Japan), isotherm of the powder was recorded, and the specific surface area of the sample was determined using BET method. With barium sulfate serving as a standard, UV-DRS measurement was performed using an UV-vis spectrophotometer (PerkinElmer, USA). As part of the analysis of the intermediates formed during the oxidation of OTC, a gas chromatography instrument with an Agilent 5973 mass spectrometer was used (Palo Alto, California).

### 2.4 Sonocatalytic process

A fixed quantity of Ti_2_SnC (1 g L^-1^) was used as the sonocatalyst in a 250 mL Erlenmeyer flask along with 100 mL of 10 mg L^-1^ OTC aqueous solution. The OTC solution was left at its normal pH for the studies (7.1). To initiate sonocatalytic degradation, the Ti_2_SnC suspension was irradiated with 36 kHz ultrasound waves that were generated by a Sonica US bath (150 W, Ultra 8060, England). At regular intervals, 5 mL of the OTC solution was collected, filtered through a 0.22-µm syringe filter, and analyzed using an UV-vis spectroscopy to determine the remained concentration of pollutants in the reactor. At a maximum wavelength of 370 nm, the UV-vis absorption of OTC was measured using a UV-vis spectrophotometer (Specord 250, Analytik Jena, Germany), and subsequently DE (%) was calculated. Experiments were conducted to determine the optimal degradation efficiency under several experimental conditions, including catalyst dosage, initial OTC concentration, pH values, and radical quenching species. The catalyst used in the recycling experiments was thoroughly rinsed using distilled water and then dried at 65 °C for 12 h between cycles.

### 2.5 Electrochemical measurement

The MAX phase was dispersed in the NMP with a concentration of 2 mg mL^−1^. Then, Nafion binder (10 μL) was added to the mixture. A well-dispersed solution was obtained by ultrasonicating the mixture for 90 min preceding being spin-coated over a graphite electrode. Subsequently, 30 μL of the solution was spin coated over a graphite electrode and dried at 80 °C. Under a three-electrode setup at room temperature and with 0.5 mol L^-1^ Na_2_SO_4_ as the electrolyte, a Mott-Schottky (M−S) plot was obtained at 1 kHz using a potentiostat-galvanostat instrument (OrigaFlex OGF01A, France). For the counter and reference electrodes, a platinum electrode and saturated calomel electrode (SCE) were employed, respectively. The working electrode was a graphite covered with the Ti_2_SnC MAX phase.

## 3 Results and discussion

### 3.1 Material characterization

**Fig. 1:**
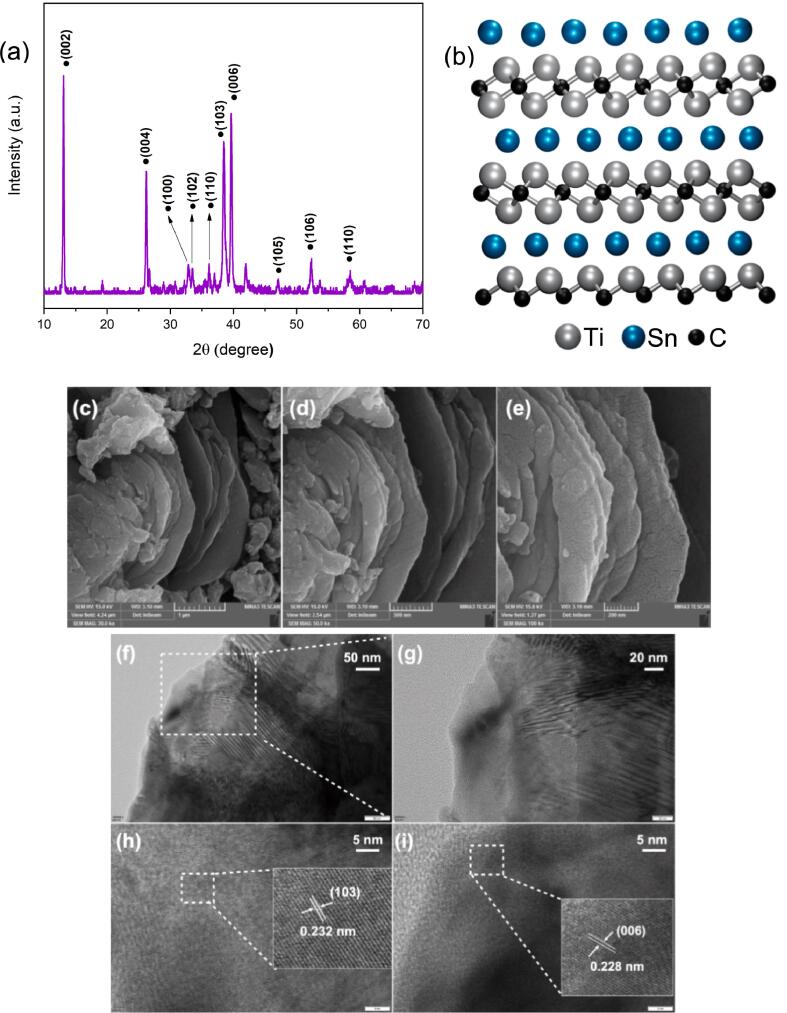
(a) XRD pattern, (b) atomic structure model, (c-e) SEM images, and (f-i) TEM images of Ti_2_SnC MAX phase.

Fig. 1 illustrates the XRD pattern, SEM, and TEM images of the Ti_2_SnC MAX phase. Based on the XRD pattern (Fig. 1a), Ti_2_SnC was successfully synthesized by the reactive sintering method. The hexagonal lattice of Ti_2_SnC is confirmed by the detected diffraction peaks at 2θ = 12.9°, 26.0°, 32.7°, 33.3°, 36.0°, 38.35°, 39.5°, 46.9°, 52.1°, and 58.3°, which correspond to (0 0 2), (0 0 4), (1 0 0), (1 0 2), (1 1 0), (1 0 3), (0 0 6), (1 0 5), (1 0 6), and (1 1 0) reflection plans, respectively [27]. In accordance with Debye-Scherrer formula [29], [30], the average crystallite size of the synthesized Ti_2_SnC was calculated to be 25 nm. In addition, the XRD pattern was utilized in conjunction with Eq. (1) to assess the MAX phase crystallinity [31]:

(1) $$Crystallinity\;\left(\%\right)\;=\frac{\text{Area of crystalline peak}}{\text{Area of all peaks}}\times 100$$

As a crucial metric, the full width at half maximum (FWHM) of a diffraction peak quantifies the material’s crystallinity. The crystallinity for the Ti_2_SnC MAX phase is determined to be 79 %.

FESEM was used for further analysis. The layered structure of Ti_2_SnC is shown in Fig. 1b, with two distinct alternating layers of Ti_2_C and Sn. The Ti_2_SnC displays a prevalent layered structure of MAX phases [7], [32], as seen in Fig. 1c–e. Also, the HRTEM images identify a layered morphology (Fig. 1f–i), with d spacings of about 0.232 and 0.228 nm, which are related to the (1 0 3) and (0 0 6) lattice planes, respectively [27], [33].

**Fig. 2:**
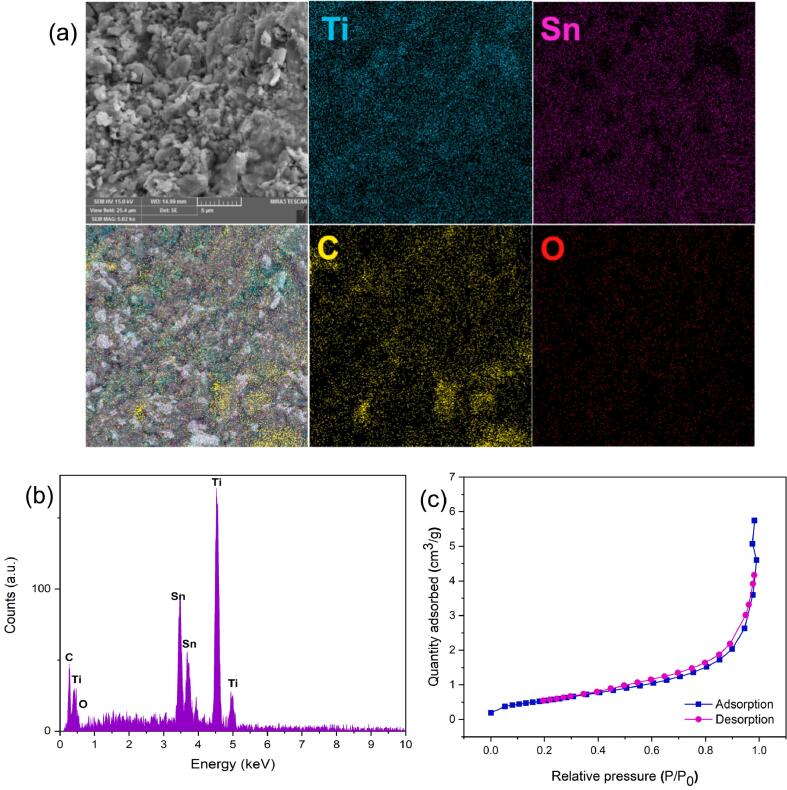
(a) SEM image and SEM elemental-mapping; (b) EDX spectrum; and (c) N_2_ adsorption–desorption isotherm of Ti_2_SnC MAX phase.

Using the elemental mapping images (Fig. 2a), it is evident that Ti, Sn, and C are homogeneously distributed throughout the Ti_2_SnC MAX phase. The EDX spectrum confirms the as-prepared MAX phase's superb purity by detecting the Ti, Sn, C, and O, with no traces of any other elements (Fig. 2b).

The nitrogen adsorption/desorption isotherm is illustrated in Fig. 2c. The Ti_2_SnC powder, measured by multipoint BET method, has a specific surface area (S_BET_) of 1.95 m^2^ g^-1^. The yielded MAX phase features a Type III isotherm, according to IUPAC classification, which indicates the nonporous structure [34].

Analyzing the UV-vis absorption spectrum of a catalyst provides information about its light-harvesting capability. The Ti_2_SnC MAX phase displays a maximum absorption near the UV range at around 240 nm (Fig. S1). The Kubelka-Munk functions represented in Eqs. (1), (2) used for calculating the catalyst band-gap energy [35]:

(2) $$F\;\left(R_{\infty \;}\right)=\frac{{\left(1-\;R_{\infty }\right)}^{2}}{2R_{\infty }}$$

(3) $${\left(F\;\left(R_{\infty \;}\right)hv\right)}^{2}=C\;\left(\mathrm{hv}-E_{g\;}\right)$$

where F is the Kubelka-Munk function, R represents diffuse reflectance, and h, υ, C, and E_g_ denote the sample's Plank constant, light frequency constant, and band-gap, respectively. By extrapolating the linear component of ${\left(F\;\left(R_{\infty \;}\right)hv\right)}^{2}$ vs energy (hѵ), 5.34 eV was determined to be the sample's band gap (Fig. S2). Sonocatalytic systems generate sonoluminescence with an energy of 6 eV [36], [37]; hence, electron-hole pairs can be formed by ultrasonic irradiation in the Ti_2_SnC MAX phase [38], which has a wide band-gap.

**Fig. 3:**
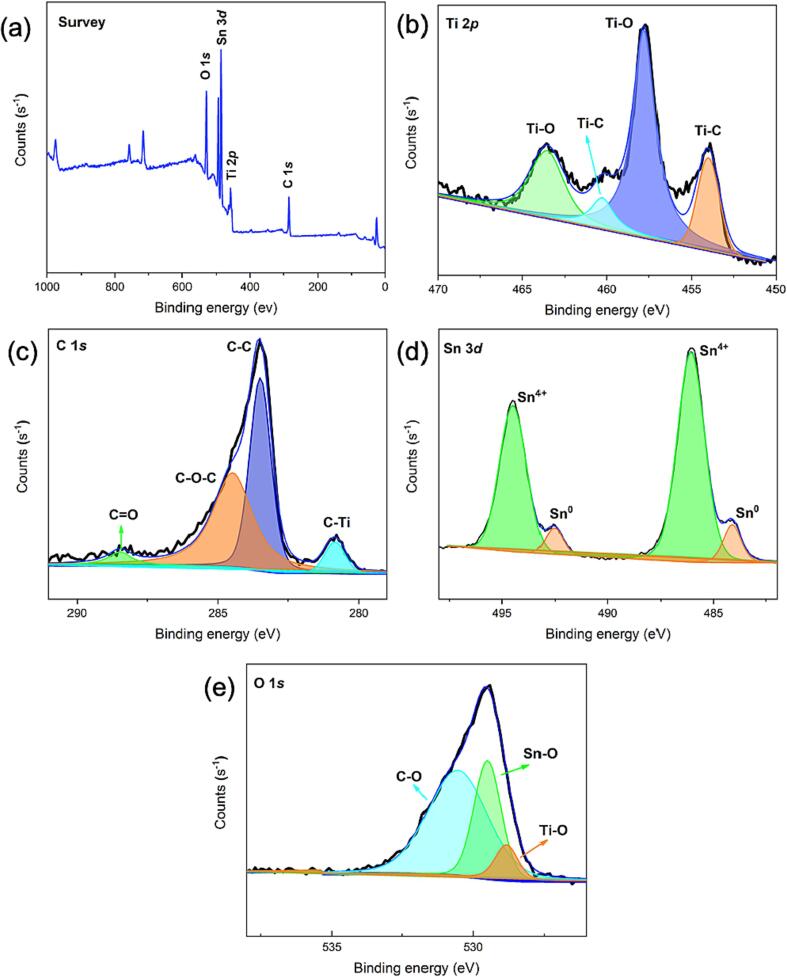
XPS spectra of (a) survey; (b) Ti 2p; (c) C 1 s; (d) Sn 3d; and (e) O 1 s in Ti_2_SnC MAX phase.

Employing XPS, the precise surface chemistry and elemental states of the Ti_2_SnC MAX phase were analyzed, and the findings are represented in Fig. 3. The MAX phase structure contains Ti, C, Sn, and O elements, as represented by the survey spectrum in Fig. 3a. According to the findings of several research on the Ti 2p deconvolution [1], [39], [40], [41], [42], the typical Ti 2p peak is consist of two doublets that correspond to Ti 2p_3/2_ and Ti 2p_1/2_ (Fig. 3b). The two peaks located at 454.0 eV (Ti 2p_3/2_) and 460.3 eV (Ti 2p_1/2_) are attributed to Ti-C, while Ti-O is represented by the peaks positioned at 457.8 eV (Ti^4+^ 2p_3/2_) and 463.6 eV (Ti^4+^ 2p_1/2_), which primarily represent the Ti_2_SnC's oxygen-terminated surface [1], [42].

Fig. 3c depicts the deconvoluted C1s spectrum of the Ti_2_SnC, which reveals four peaks. The bonding energies attributed to C-Ti, C—C, C—O—C, and C=O are reflected at 280.8, 283.4, 284.4, and 288.5 eV, respectively [32], [43]. The high-resolution Sn 3d spectrum of Ti_2_SnC is shown in Fig. 3d. Briefly, the as-prepared Ti_2_SnC MAX phase features Sn^0^ (493.79 and 485.35 eV) and Sn^4+^ (495.8 and 487.3 eV) peaks [27], [44]. While the Sn^4+^ peak in the sample suggests surface oxidation; the Sn^0^ peak indicates the presence of closely packed nanosheets. The O 1 s spectra (Fig. 3e) demonstrates three peaks at 528.8, 529.5, and 530.5 eV, which are ascribed to TiO_2_, SnO_2_, and C—O bonds, respectively [27]. All of the aforementioned data verifies the highly pure Ti_2_SnC synthesis.

### 3.2 Sonocatalytic activity of Ti_2_SnC

**Fig. 4:**
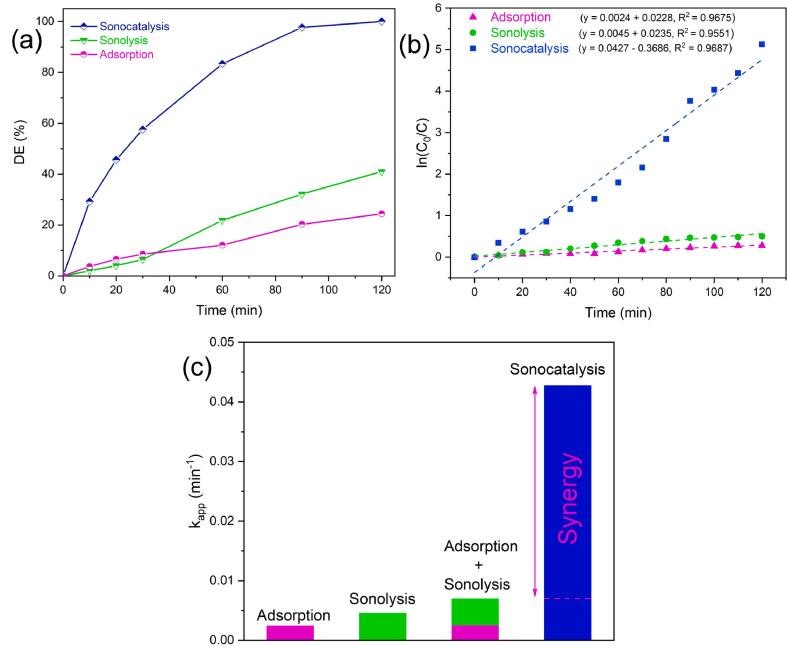
(a) Degradation efficiency of OTC via varied systems; (b) the pseudo-first-order kinetic rate plots for sonocatalytic process; and (c) synergy factor assessment for the sonocatalytic process. Operational condition: [Ti_2_SnC] = 1 g L^-1^, [OTC]_0_ = 10 mg L^-1^, and natural pH.

In this research, OTC was used as a model antibiotic pollutant to assess the sonocatalytic activity of Ti_2_SnC MAX phase. For the candidate pollutant, we designed a series of tests in which Ti_2_SnC was investigated to establish the desired working parameters for the OTC sonocatalytic degradation (e.g., catalyst dosage, pH, and OTC concentration). This study aimed to assess the synergistic effects of three significant factors: sonolysis (individually exposing the OTC solution to the US waves), adsorption (a solution incorporating Ti_2_SnC and OTC), and sonocatalysis (the combination of Ti_2_SnC with the US waves). On its own, the sonolysis was only able to break down 39.4 % of OTC (10 mg L^-1^), as seen in Fig. 4a. Therefore, the sonolysis is inefficient because it does not produce a significant number of reactive radicals. The adsorption capability of Ti_2_SnC was next assessed by adding 1 g L^-1^ Ti_2_SnC to a 10 mg L^-1^ OTC solution and stirring the mixture in the dark for 2 h, during which 21.3 % of OTC was removed. Finally, the sonocatalysis strategy achieved the complete degradation (100 %) of OTC under the desired operational condition. The elevated degradation efficiency of OTC in the presence of the Ti_2_SnC catalyst is due to accelerated cavitation phenomena, which results in more cavitation bubbles [45]. The microbubbles finally collapse and generate a considerable amount of localized energy and sonoluminescence, which can form electron hole pairs on the sonocatalyst. Following that, the in-situ-generated ROS can attack and oxidize the pollutants to intermediates and final mineralization products of H_2_O and CO_2_ [46].

A synergy index quantifies the synergistic impact. The index over 1.00 indicates increased efficiency in coupling chosen techniques than comparing them independently [47]. For the purpose of ascertaining the synergy, pseudo-first-order kinetic model can be used. After calculating the estimated pseudo-first-order rate constant (k_app_) one per system (Fig. 4b), the synergy index is computed as Eq. (4) [48]:

(4) $$\text{Synergy index =}\;\frac{k_{\mathrm{app}}\left(sonocatalysis\right)}{k_{\mathrm{app}}\left(sonolysis\right)+k_{\mathrm{app}}\left(adsorption\right)}$$

The k_app_ for the sonocatalysis, sonolysis, and adsorption was determined to be 0.0427, 0.0045, and 0.0024 min^−1^. The synergy index of 6.1 is derived as a consequence of coupling the sonocatalyst and the sonolysis process, suggesting a significant degree of synergy (Fig. 4c). As a simple method of assessing catalytic degradation systems, degradation turnover (dTON) has recently been proposed as a numerical metric. This value allows catalysts to be compared despite the amounts of contaminants and catalysts. The following is a formula for computing the dTON, Eq. (5) [49]:

(5) $$\text{dTON =}\;\frac{\left[Mi]-[Mf\right]}{t\times [Cat.]}$$

Assuming that M_i_ and M_f_ are the pre-treatment and post-treatment concentrations of the contaminant (µM), t denotes time (h), and Cat signifies the quantity of the catalyst (g L^-1^). The dTON of the sonocatalytic experiment is estimated to be 10.8 µmol h^−1^ g_cat_^-1^. As shown in Table S1, the dTON of previous catalytic systems are provided.

#### 3.2.1 Sonocatalysis parameters

**Fig. 5:**
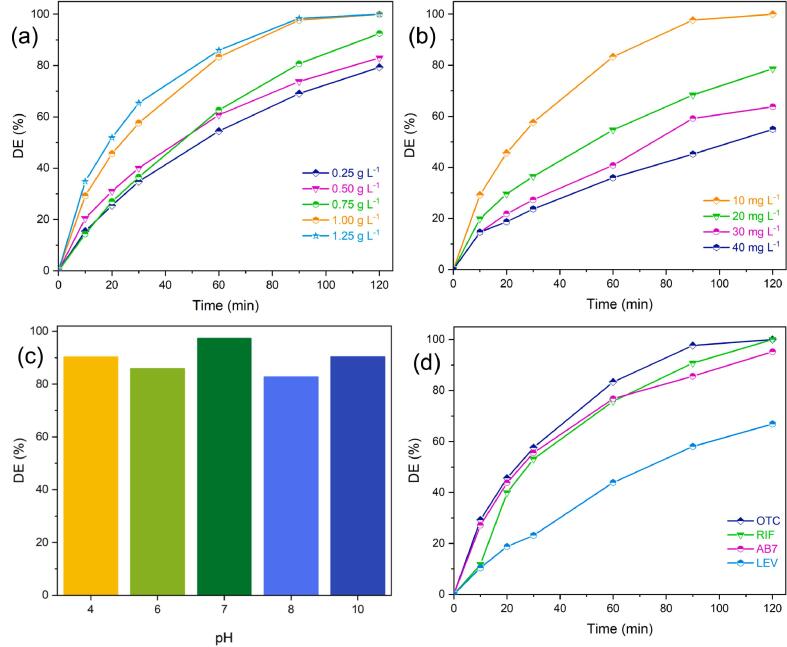
Effects of (a) Ti_2_SnC MAX phase dosage, (b) OTC concentration, (c) various pH on OTC sonocatalytic degradation during 90 min of reaction time, and (d) removal of different contaminants. Operational conditions: [Ti_2_SnC] = 1 g L^-1^, [OTC]_0_ = [RIF]_0_ = [AB7]_0_ = [LEV]_0_ = 10 mg L^-1^, and natural pH.

The first variable to be investigated in the sonocatalytic degradation of OTC was the amount of catalyst loading, which has a significant impact on DE (%). Fig. 5a shows the sonocatalytic degradation of the OTC-contaminated aqueous solution (10 mg L^-1^) when a set of catalyst doses ranging from 0.25 to 1.25 g L^-1^ was used. By increasing the catalyst dosage from 0.25 to 1 g L^-1^, the DE (%) increases from 79.3 % to 100 %. Accordingly, adjusting the catalyst amount to 1.25 g L^-1^ has a negligible effect on the complete degradation rate. This prompted the selection of 1 g L^-1^ as the ideal catalyst dosage for a cost-effective sonocatalytic system. Escalated DE (%) at increased catalyst dosages is explained by the existence of more active sites that would accelerate the formation of reactive species [50]. Amplified absorption of photon energy by the surface of Ti_2_SnC is responsible for this more active sites imply more reactive species, which accelerates the oxidation pathway toward OTC degradation.

The DE (%) was evaluated with OTC doses ranging from 10 to 40 mg L^-1^. Even in trace amounts, emerging contaminants' residues in the environment have adverse effects on the existing ecosystem and human health [51]. As shown in Fig. 5b, low concentrations result in higher degradation. By increasing the OTC concentration to 30 and 40 mg L^-1^, DE (%) is reduced to 63.7 % and 54.9 %, respectively. Aside from the sonocatalyst's limited degradation ability, OTC molecules accumulate on the Ti_2_SnC surface, inhibiting sonocatalyst surface activity, and hence impeding OTC degradation [52]. To be more specific, as OTC concentration rises

(i) more intermediates are formed during sonocatalytic degradation, and these compounds can be adsorbed on the active sites of the Ti_2_SnC MAX phase, preventing the catalyst's ability to absorb heat and cavitation bubble energy, and (ii) rate-limited reactive species are not adequate to degrade the targeted concentration of contaminant [53].

At varying pH levels, OTC occurs in cationic (H_3_OTC^+^), zwitterionic (H_2_OTC), anionic (HOTC^−^), and dianionic (OTC^2−^) forms [54]. The degradation efficiency of OTC was investigated as a function of starting pH (in the range of 4–10). There was total decomposition at all pH values within 120 min of reaction time, although it occurred at varying rates. Thus, throughout the first 90 min, the impact of initial pH was studied. The degradation efficiency of 97 % was attained at pH = 7.1 as shown in Fig. 5c. The DE (%) was seen to decrease both above and below the optimal pH (7.1). A soncatalyst's point of zero charge (pH_pzc_) value provides insight into the observed trend. Considering Fig. S2, we can see that at room temperature, the pH_pzc_ of Ti_2_SnC is 6.77. At pH levels above pH_pzc_, hydroxide anions are adsorbed onto the sonocatalyst, giving its surface a negative charge. However, for pH levels below pH_pzc_, protons are adsorbed onto the surface of Ti_2_SnC, leading to a positive charge. As a result of deprotonation, the OTC molecules become negatively charged at a pH greater than 7.3 (pKa_2_ of OTC) [55]. The negative charge of the sonocatalyst and OTC causes them to repel one another due to electrostatic repulsion, which contributes to the drop in DE (%) seen at pH values over 7.1. However, at an acidic pH, the OTC molecules become positively charged, which is attributed to protonation. Therefore, the decreased DE (%) seen at acidic pHs can be traced, in large part, to the electrostatic repulsion between the protonated OTC molecules and the positively charged sonocatalyst.

The effectiveness of various sonocatalysts in degrading tetracycline antibiotics is compared with the present work and summarized in Table S2.

#### 3.2.2 Degradation of various organic pollutants

The effectiveness of the proposed Ti_2_SnC sonocatalytic system was assessed on the degradation of RIF, LEV, and AB7 in addition to OTC. Discharging various pharmaceuticals and dyes into wastewater results in long-term health problems. Having antimycobacterial activity against tuberculosis, a fatal contagious ailment, RIF stops bacterial growth by hindering bacterial ribonucleic acid (RNA) synthesis [56]. As a result of its high-water solubility, RIF has attracted the attention of researchers in the field of water and wastewater treatment [57]. In addition, LEV, a broad-spectrum fluoroquinolone antibiotic, is taken into account as a pharmaceutical pollutant in the current investigation. Both the oral and intravenous forms of LEV possess remarkable bactericidal and tissue-penetrating properties. Extreme solubility in water (16.98 mg mL^−1^ at 298.15 K) means that LEV accumulates considerably in both surface and subsurface water [58]. Tragically, it has prompted serious issues, including the perpetuation of drug resistance and even the reproduction of superbacteria [59]. Nowadays, awareness of the environmental problems caused by the textile industry has been on the rise [60]. Triarylmethane dyes, such as AB7, are among the many pollutants found in wastewaters from the textile sector [61]. With a molecular weight of 690.80 g mol^−1^, AB7 is a triaryl methane dye with a very intricate structure. With a mouse LD50 of 437 mg kg^−1^, AB7 is toxic despite being extremely water-soluble anionic dye [61]. This study compared the degradation efficiency of RIF, LEV, and AB7 in the presence of 1 g L^-1^ of Ti_2_SnC when subjected to ultrasonic irradiation at an initial concentration of 10 mg L^-1^. Following 120 min of reaction time, as shown in Fig. 5d, 100 %, 95 %, and 67.3 % of the RIF, AB7, and LEV were eliminated, respectively.

#### 3.2.3 Identification of oxidizing species

**Fig. 6:**
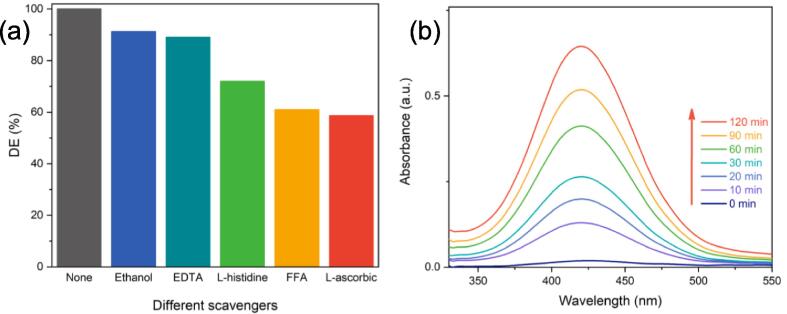
Effect of various scavengers on OTC sonocatalytic degradation; Operational conditions: [OTC:scavenger]_0_ = 1:20, [Ti_2_SnC] = 1 g L^-1^, [OTC]_0_ = 10 mg L^-1^, and natural pH and (b) UV–vis absorption spectra of o-phenylenediamine with Ti_2_SnC in deionized water under ultrasonic irradiation. Operational conditions: [Ti_2_SnC] = 1 g L^-1^, and pH = 7.

A comprehensive study was conducted to gain a deeper understanding of reactive species performance in the sonocatalytic degradation of OTC. l-histidine and furfuryl alcohol were utilized as singlet oxygen scavengers [62], [63], whereas EDTA, ethanol, and l-ascorbic acid were used to quench ^•^OH, sonogenerated holes [64], and superoxide [65], respectively. An exact molar ratio of 1:20 was used for OTC:scavenger in these experiments [66]. Adding radical and nonradical scavengers had an inhibiting effect on the DE (%), decreasing it in the sequence of O_2_^•−^ > ^1^O_2_ > h^+^ > ^•^OH (Fig. 6a). Introducing ethanol and EDTA, DE (%) declined to 91.2 and 88.9 %, respectively; the reductions, however, were not substantial. In contrast, the l-ascorbic acid, FFA, and l-histidine significantly reduced DE (%) to 58.6, 60.9, and 71.9 %, respectively. Additionally, FFA is also capable of reacting with ^•^OH. Considering this, OTC with FFA has a much lower DE (%) than with ethanol, suggesting that the singlet oxygen, in addition to the hydroxyl radicals, plays an important role in the pollutant degradation; this indicates that the formation of oxidizing species under the US irradiations is less dependent on the sonogenerated holes in Ti_2_SnC. It can also be deduced that the enhanced sonocatalytic degradation of OTC by Ti_2_SnC is due to the indirect production of ^1^O_2_ from the dissolved oxygen.

#### 3.2.4 Identification of hydroxyl radicals

To verify further, the formation of ^•^OH can be determined by sonicating an aqueous solution of Ti_2_SnC with O-phenylenediamine (OPD) at pH of 7, which is adjusted by sodium phosphate buffer. A visible spectrophotometer was used to detect the hydroxyl radicals using OPD as a probe; the o-phenylenediamine reacts with ^•^OH radical Eq. (6) to form 2,3-diaminophenazine molecule (DAPN) which is yellow in color with maximum absorption at wavelength of 420 nm [67], [68]. As can be observed in Fig. 6b, the absorption intensity and, more specifically, the ^•^OH radicals production increases with time, demonstrating the process ability to generate these radicals during the sonocatalytic process.

**(6):**
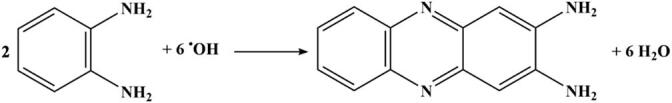

#### 3.2.5 Probable degradation mechanism and generated intermediates

**Fig. 7:**
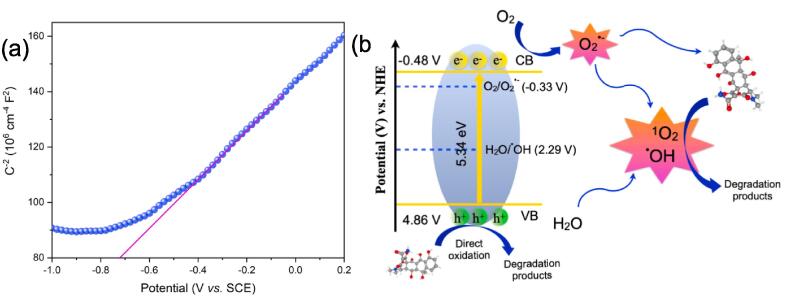
(a) The Mott − Schottky plot, and (b) band positions of the Ti_2_SnC MAX phase.

An ostensible mechanism underlying the Ti_2_SnC-mediated sonocatalytic degradation of OTC is investigated further. Sonogenerated sonoluminescence with an average energy of 6 eV [36], [37], provides the energy necessary to induce excitation in the Ti_2_SnC MAX phase, which in turn generates electrons and holes on the corresponding CB and VB. Hence, the generated electron-hole either directly degrades OTC or indirectly promotes its degradation by triggering formation of the reactive species. Generally, sonocatalytic activity is determined by the band edge energies. According to the Mott-Schottky test, the semiconductor type and flat band potential (E_fb_) can be determined [69], [70]. As shown in the Fig. 7a, the positive slope proves the *n*-type semiconducting [71], [72] nature of the Ti_2_SnC MAX phase. Based on the intercept obtained by aligning the linear portion of the M−S plot to the potential axis [73], the catalyst’s flat band potential is determined as −0.72 V vs SCE. Using the Nernst equation, E_(NHE)_ = E_(SCE)_ + 0.24 [74], the flat band potential value is adjusted with regard to the normal hydrogen electrode potential (−0.48 V vs NHE). Generally, the E_fb_ of a *n*-type semiconductor is nearly identical to its E_CB_ [75]. According to the E_g_ of the Ti_2_SnC and the band gap equation (E_CB_ = E_VB_ – E_g_) [76], the E_VB_ is calculated to be 4.86 V. As the band positions of the sample are determined (Fig. 7b), developing a mechanism to study the OTC sonodegradation is the next step.

To specify further, the Ti_2_SnC's CB is positioned negative enough to the reduction potential of O_2_ (−0.33 V vs NHE) [77], which makes it possible to generate O_2_^•−^ through the reaction with the dissolved oxygen Eq. (9). Consequently, the generated O_2_^•−^ is converted into ^1^O_2_ at the holes in the Ti_2_SnC valence band, Eq. (10) [72]. These reactive species are predominant in the sonodegradation process, confirmed by scavengers' findings. As well as that, the holes formed in the VB can directly oxidize OTC [78]. Aside from the sonolysis pathway, Eq. (7), the aforementioned findings indicate that the Ti_2_SnC MAX phase is capable of ^•^OH formation, Eq. (8). Accordingly, in the Ti_2_SnC sonocatalytic system, the active oxidizing species O_2_^•−^, ^1^O_2_, and ^•^OH in addition to sono-induced h^+^ are responsible for generating the remarkable sonocatalytic activity. Eventually, the OTC mineralization is possible through the further oxidization of smaller degradation intermediates, Eq. (11).

(7) H_2_O + ))) → ^•^H + ^•^OH

(8) H_2_O + h^+^ →^•^OH + H^+^

(9) Ti_2_SnC (e^-^) + O_2_ → Ti_2_SnC + O_2_^•−^

(10) Ti_2_SnC (h^+^) + O_2_^•−^→ Ti_2_SnC + ^1^O_2_

(11) OTC + O_2_^•−^/^1^O_2_/h^+^/^•^OH → degradation products

**Scheme 1:**
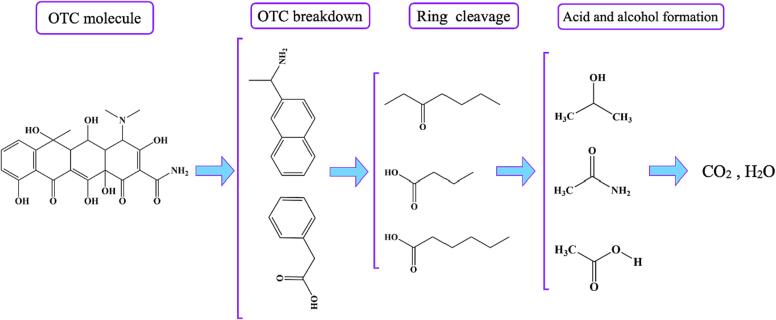
Mineralization of OTC over the Ti_2_SnC MAX phase.

In an attempt to determine the intermediates formed by the sonocatalytic decomposition of OTC through the Ti_2_SnC MAX phase, the processed solution was subjected to GC–MS analysis. As a result of fast oxidation during the sonocatalytic process, identifying all the sonogenerated intermediates may not be feasible. Eight plausible main intermediates deriving from OTC degradation are listed in Table S3 along with their chemical composition and molecular formula. Scheme 1 depicts the formation of short chains from ring-containing intermediates when OTC is degraded by Ti_2_SnC sonocatalysis. The mentioned ROS attack the OTC molecules, breaking their C—C, C=C, C—N, C—O, and C=O bonds. Hence, the aromatic rings can be directly oxidized, producing smaller intermediates, as evidenced by the presence of several molecules in the solution. According to Scheme. 1, the majority of the generated intermediates are innocuous molecules, including acetic acid and propanol molecules.

#### 3.2.6 Real water samples and reusability potential

**Fig. 8:**
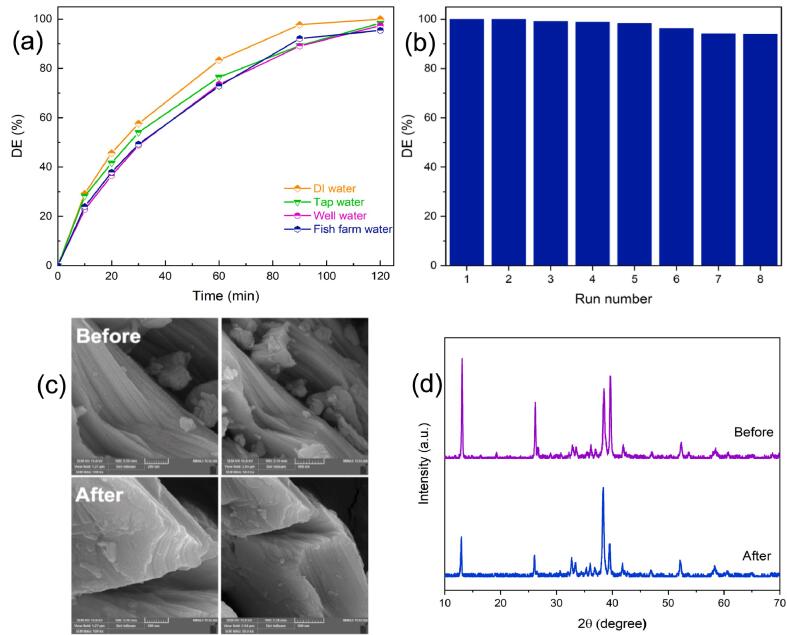
(a) Examining the sonocatalytic degradation of OTC by Ti_2_SnC in practical water matrices, i.e., well, fish farm, and tape water; (b) recyclability of Ti_2_SnC MAX phase. Operational conditions: [Ti_2_SnC] = 1 g L^-1^, [OTC]_0_ = 10 mg L^-1^, and natural pH; (c) SEM images, and (d) XRD patterns of Ti_2_SnC MAX phase before and after OTC sonocatalytic degradation.

Having achieved the proper outcomes from the OTC solution in distilled water (DI), OTC was added to actual water samples at a determined concentration (i.e., well water and tap water; Table S4 provides characteristics of real water samples). To examine sonocatalytic performance, the desired conditions were replicated in the lab and applied to real samples. Fig. 8a depicts the influence of various water sources on the sonocatalytic degradation of OTC. The degradation efficiency of OTC in tap water, well water, and fish farms was observed to be 98.3 %, 97.3 %, and 95.4 %, respectively. Based on these experimental findings, Ti_2_SnC MAX phase can be used in real water matrices as well.

The reusability test appears to be vital in evaluating the efficacy of catalysts in treating industrial effluent [79], [80]. After each run of the reuse test, the catalyst was centrifuged out of the solution and washed with deionized water before being used again. Similar optimum conditions were applied to the same Ti_2_SnC sample during eight sequential cycles of the OTC sonocatalytic degradation. As indicated in Fig. 8b, DE (%) decreases by just 6.0 % after eight runs. Besides, the catalyst's structural stability was investigated using the XRD and FESEM analyses after the eight runs (Fig. 8c, d). The XRD patterns and morphology of the fresh catalyst are strikingly similar to those of the used catalyst, confirming that the Ti_2_SnC MAX phase is structurally stable throughout the successive applications. According to the results, the synthesized MAX phase offers promising structural stability and adequate reusability for removing antibiotics.

## 4 Conclusion

A novel nano-laminated sonocatalyst, the Ti_2_SnC MAX phase, was synthesized through the reactive sintering approach and evaluated for its sonocatalytic activity toward degrading OTC, an identified emerging contaminant. The XRD pattern verified the successful synthesis of the Ti_2_SnC MAX phase in a hexagonal crystal lattice. Closely packed layered morphology was observed for the outlined titanium-based MAX phase by FESEM and HRTEM. Further characteristics were obtained through XPS, EDX, BET, and UV-DRS analyses, which also confirmed the successful formation of Ti_2_SnC MAX phase. Through the degradation of OTC, the sonocatalytic activity of the as-prepared MAX phase was evaluated, with the goal of lowering the potential pollution caused by emerging contaminants. The ultimate degradation efficiency of 100 % has been achieved, under the desired operational conditions in 2 h ([Ti_2_SnC] = 1 g L^-1^, [OTC] = 10 mg L^-1^, and natural pH). The Ti_2_SnC MAX phase demonstrated remarkable sonocatalytic activity, even in the actual water samples, along with its superior stability during ultrasonic irradiation after eight runs. Degradation of RIF, AB7, and LEV was also effectively accomplished by the Ti_2_SnC-mediated sonocatalytic system. Studies involving the Mott-Schottky measurements and the ROS scavengers have provided insight into the sonocatalytic degradation mechanism of OTC, suggesting the predominant role of both singlet oxygen and superoxide ions. According to the GC–MS analysis, a plausible mechanism was proposed. Eventually, it has been demonstrated that the MAX phase-based materials possess strong potential as sonocatalysts for degrading antibiotic contaminants in water matrices.

## CRediT authorship contribution statement

**Samira Haddadi:** Investigation, Writing – original draft. **Alireza Khataee:** Supervision, Writing – review & editing. **Samira Arefi-Oskoui:** Conceptualization, Writing – review & editing. **Behrouz Vahid:** Writing – review & editing. **Yasin Orooji:** Writing – review & editing. **Yeojoon Yoon:** Writing – review & editing.

## Declaration of Competing Interest

The authors declare that they have no known competing financial interests or personal relationships that could have appeared to influence the work reported in this paper.

## Data Availability

No data was used for the research described in the article.
