# Supplementary material for: Titanium-based MAX-phase with sonocatalytic activity for degradation of oxytetracycline antibiotic
Source: Ultrason Sonochem. 2022 Dec 5;92:106255. doi: 10.1016/j.ultsonch.2022.106255 (PMC9763513; doi:10.1016/j.ultsonch.2022.106255)
Supplement: Supplementary data 1 [file mmc1.docx]

**Supplementary information**

**Titanium-based MAX-phase with sonocatalytic activity for degradation of oxytetracycline antibiotic**


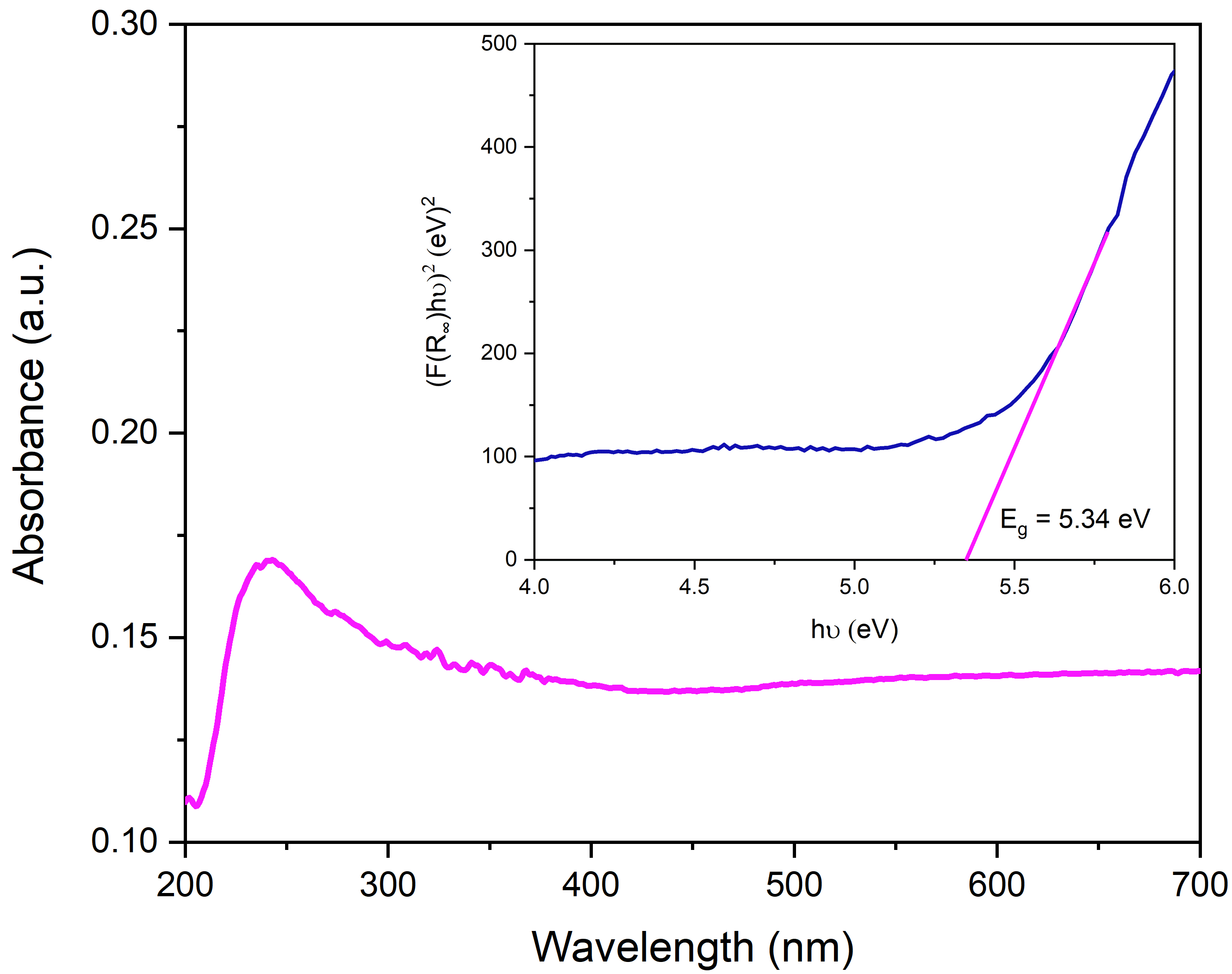


**Figure S1.** DRS spectrum, and Tauc plot (inset) of the Ti_2_SnC MAX phase.


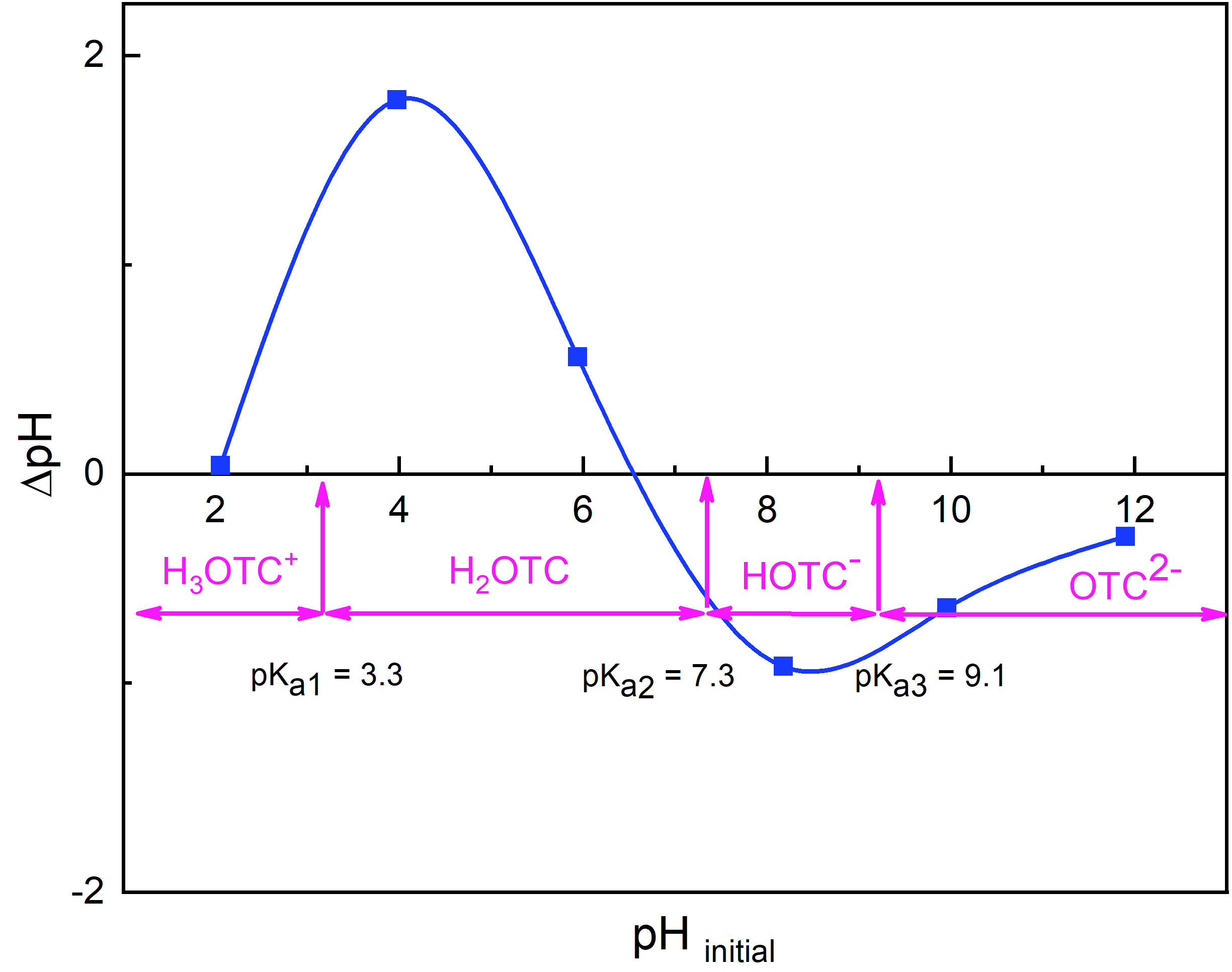


**Figure S2.** pH variation during the sonocatalytic degradation through Ti_2_SnC versus the initial pH of the treatment.

**Table S1.** dTON for different AOPs.

| **Catalyst** | **AOP method** | **pollutant** | **Catalyst dosage**  **(g/L)** | **Pollutant concentration**  **(mg/L)** | **Reaction time**  **(h)** | **Degradation**  **Efficiency**  **(%)** | **dTON**  **(μmol/h g)** | **Ref** |
| --- | --- | --- | --- | --- | --- | --- | --- | --- |
| CU-THAp | Catalyst/PDS | RhB | 1 | 20 | 2 | 78.28 | 2.05 | [[1](#_ENREF_1)] |
| ZnCoFe LDH | Photocatalysis | MB | 0.1 | 2.5 | 3 | 74 | 24.49 | [[2](#_ENREF_2)] |
| Truncated octahedral Cu_2_O | Photocatalysis | TC-HCl | 0.1 | 20 | 1 | 31.67 | 54.98 | [[3](#_ENREF_3)] |
| Octahedral Cu_2_O | Photocatalysis | TC-HCl | 0.1 | 20 | 1 | 31.33 | 66.68 | [[3](#_ENREF_3)] |
| Ti_2_SnC MAX phase | Sonocatalysis | OTC | 1 | 10 | 2 | 100 | 10.8 | **This work** |

| Ref | Degradation efficiency (%) | Time (min) | Sonocatalysis condition | Tetracyclines concentration (mg L^-1^) | Sonocatalyst amount  (g L^-1^) | Sonocatalyst |
| --- | --- | --- | --- | --- | --- | --- |
| [[4](#_ENREF_4)] | 75.3 | 120 | US power: 500 W | 25 | 1 | 0.6Ag_3_PO_4_/CoWO_4_ |
| [[5](#_ENREF_5)] | 79 | 300 | US power: 300 W, pH: 6 | 10 | 1 | SrTiO_3_/Ag_2_S/CoWO_4_ |
| [[6](#_ENREF_6)] | 95 | 70 | US power: 200 W, pH: 7, Na_2_S_2_O_8_: 1 g L^-1^ | 2 | 3 | Flower-like ZnO |
| [[7](#_ENREF_7)] | ~93 | 70 | US power: 400 W | 10 | 0.5 | BiOI microsphere |
| [[8](#_ENREF_8)] | 30 | 60 | US power: 50 W, pH: natural pH of TC | 25 | 0.6 | Mn-modified Diatomite |
| [[9](#_ENREF_9)] | 65 | 120 | US power: 500 W, pH: 4 | 25 | 0.25 | TiO_2_ NPs (P25) |
| This work | 100 | 120 | US power: 150 W, pH: natural pH of OTC | 10 | 1 | Ti_2_SnC MAX phase |

**Table S2.** Comparison of different sonocatalysts for the tetracycline antibiotics degradation.

| No. | Compound Name | Structure | t_R_(min) | Main fragments (m/z)/(percent) |
| --- | --- | --- | --- | --- |
| 1 | Acetamide | 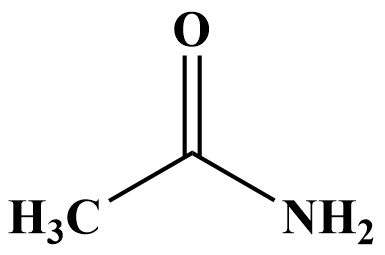 | 3.47 | 59.10 (100.00%), 60.00 (3.26%), 147.00 (2.37%), 75.00 (0.85%), 73.00 (0.77%) |
| 2 | Acetic acid |  | 4.715 | 75.00 (100.00%), 116.10 (94.47%), 73.10 (14.23%), 117.00 (12.79%), 76.00 (10.42%) |
| 3 | 2-Propanol | 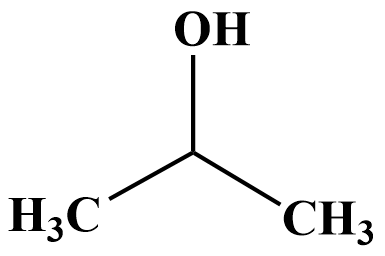 | 23.934 | 72.10 (100.00%), 73.00 (24.07%), 117 (14.33%), 75.00 (12.39%), 313.30 (10.18%) |
| 4 | Butanoic acid | 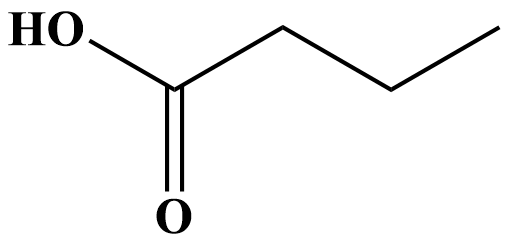 | 5.614 | 75.00 (100.00%), 116.10 (94.47%), 73.10 (14.23%), 117.00 (12.79%), 76.00 (10.42%) |
| 5 | Hexanoic acid | 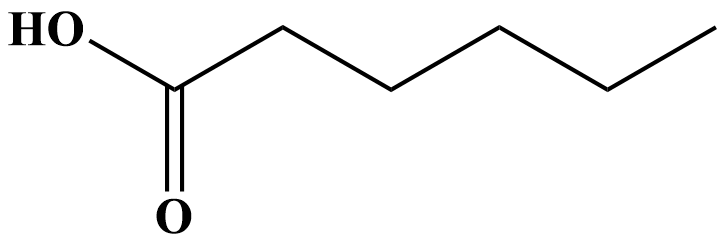 | 6.46 | 73.00 (100.00%), 75.00 (99.67%), 59.00 (83.85%), 221.10 (66.65%), 173.10 (63.36%) |
| 6 | 3-Heptanone | 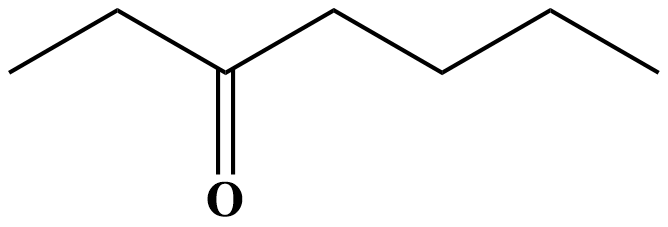 | 22.647 | 72.10 (100.00%), 73.10 (34.78%), 75.00 (18.69%), 117.00 (15.98%), 313.20 (12.49%) |
| 8 | Phenylacetic acid | 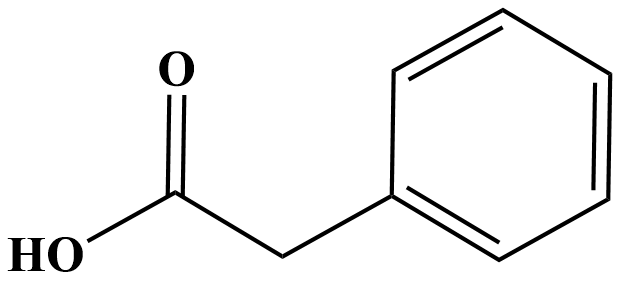 | 31.92 | 72.10 (100.00%), 131.00 (38.34%), 73.00 (22.14%), 57.10 (19.05%), 55.00 (18.78%) |
| 9 | 1-(2-Naphthyl) ethanamine | 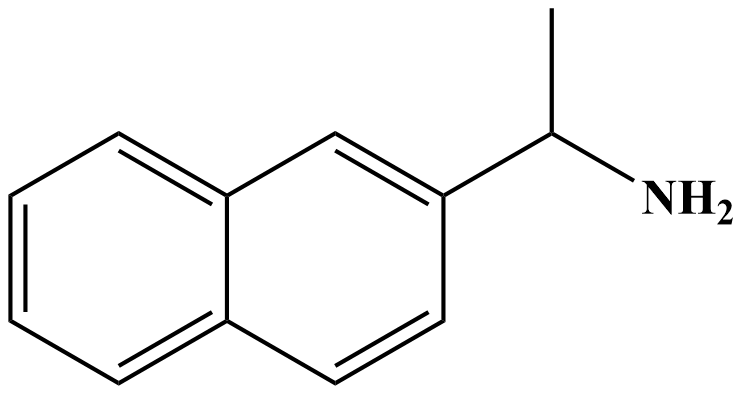 | 31.08 | 72.00 (100.00%), 57.10 (36.99%), 71.10 (24.04%), 73.00 (22.71%), 55.10 (17.59%) |

**Table S3.** GC-MS identified intermediated during OTC sonocatalytic degradation over the Ti_2_SnC.

| Tap water | Well water | Fish-farm water | parameter |
| --- | --- | --- | --- |
| 602 | 685 | 958 | **E.C.** (µS/cm) |
| 6.73 | 7.59 | 7.76 | **pH** |
| 435.29 | 612 | 859 | **TDS** (mg/L) |
| 2.63 | 2.19 | 2.27 | **Turbidity** (NTU) |
| 281.51 | 451.34 | 439.58 | **Total hardness** (mg/L CaCO_3_) |
| 77.85 | 81.45 | 99.86 | **Alkalinity** (mg/L CaCO_3_) |
| 5.47 | 365.74 | 27.43 | **K^+^** (mg/L) |
| 36.22 | 55.34 | 116.12 | **Na^+^** (mg/L) |
| 76.18 | 148.89 | 110.31 | **Ca^2+^** (mg/L) |
| 26.71 | 19.57 | 42.73 | **Mg^2+^** (mg/L) |
| - | - | 2.35 | **NH_4_^+^** (mg/L) |
| 0.49 | 0.35 | 0.17 | **F^-^** (mg/L) |
| 81.02 | 62.46 | 98.11 | **Cl^-^** (mg/L) |
| 12.48 | 19.97 | 19.02 | **NO_3_^-^** (mg/L) |
| 116.69 | 105.66 | 75.16 | **SO_4_^2-^** (mg/L) |

**Table S4.** Characterization of real water samples.

**Reference:**

[1] F. Rahmani, A. Ghadi, E. Doustkhah, S. Khaksar, In Situ Formation of Copper Phosphate on Hydroxyapatite for Wastewater Treatment, Nanomaterials 12 (2022) 2650-2660. <https://doi.org/10.3390/nano12152650>.

[2] R. Keyikoğlu, I.N. Doğan, A. Khataee, Y. Orooji, M. Kobya, Y. Yoon, Synthesis of visible light responsive ZnCoFe layered double hydroxide towards enhanced photocatalytic activity in water treatment, Chemosphere 309 (2022) 136534-136541. <https://doi.org/10.1016/j.chemosphere.2022.136534>.

[3] Y. Wu, Y. Li, H. Li, H. Guo, Q. Yang, X. Li, Tunning heterostructures interface of Cu_2_O@ HKUST-1 for enhanced photocatalytic degradation of tetracycline hydrochloride, Sep. Purif. Technol. 303 (2022) 122106-122115. <https://doi.org/10.1016/j.seppur.2022.122106>.

[4] X. Wang, N.P. Liu, H.L. An, W.T. Ju, B. Liu, X.F. Wang, L. Xu, Preparation of Ag_3_PO_4_/CoWO_4_ S-scheme heterojunction and study on sonocatalytic degradation of tetracycline, Ultrason. Sonochem. (2022) 106147-106159. <https://doi.org/10.1016/j.ultsonch.2022.106147>.

[5] J. Qiao, H. Zhang, G. Li, S. Li, Z. Qu, M. Zhang, J. Wang, Y. Song, Fabrication of a novel Z-scheme SrTiO_3_/Ag_2_S/CoWO_4_ composite and its application in sonocatalytic degradation of tetracyclines, Sep. Purif. Technol. 211 (2019) 843-856. <https://doi.org/10.1016/j.seppur.2018.10.058>.

[6] A.K. Subramani, P. Rani, P.-H. Wang, B.-Y. Chen, S. Mohan, C.-T. Chang, Performance assessment of the combined treatment for oxytetracycline antibiotics removal by sonocatalysis and degradation using Pseudomonas aeruginosa, J. Environ. Chem. Eng. 7 (2019) 103215-103225. <https://doi.org/10.1016/j.jece.2019.103215>.

[7] L. Zhong, C. Wang, X. Cui, Use of mesoporous BiOI microspheres for sonocatalytic degradation of tetracycline hydrochloride, Ecotoxicol. Environ. Saf. 237 (2022) 113547-113554. <https://doi.org/10.1016/j.ecoenv.2022.113547>.

[8] Y. Guo, X. Mi, G. Li, X. Chen, Sonocatalytic degradation of antibiotics tetracycline by Mn-modified diatomite, J. Chem. 2017 (2017). <https://doi.org/10.1155/2017/2830138>.

[9] M. Hoseini, G.H. Safari, H. Kamani, J. Jaafari, M. Ghanbarain, A.H. Mahvi, Sonocatalytic degradation of tetracycline antibiotic in aqueous solution by sonocatalysis, Toxicol. Environ. Chem. 95 (2013) 1680-1689. <https://doi.org/10.1080/02772248.2014.901328>.
